# Development and validation of a pretreatment nomogram to predict overall survival in gastric cancer

**DOI:** 10.1002/cam4.3225

**Published:** 2020-06-26

**Authors:** Etsuro Bando, Xinge Ji, Michael W. Kattan, Ho Seok Seo, Kyo Young Song, Cho‐Hyun Park, Maria Bencivenga, Giovanni de Manzoni, Masanori Terashima

**Affiliations:** ^1^ Division of Gastric Surgery Shizuoka Cancer Center Shizuoka Japan; ^2^ Department of Quantitative Health Sciences The Cleveland Clinic Cleveland OH USA; ^3^ Division of Gastrointestinal Surgery Department of Surgery Seoul St. Mary’s Hospital College of Medicine The Catholic University of Korea Seoul Republic of Korea; ^4^ Division of General and Upper Gastrointestinal Surgery Department of Surgery University of Verona Verona Italy

**Keywords:** clinical staging, gastric cancer, pretreatment nomogram, the American Joint Committee on Cancer

## Abstract

**Background:**

Pretreatment clinical staging is essential to select therapy. However, there have been no published pretreatment gastric cancer nomograms constructed using pretreatment clinical prognostic factors, including in nonresection patients. We aimed to develop a new pretreatment gastric cancer nomogram for individualized prediction of overall survival (OS).

**Methods:**

The nomogram was developed using data of 5231 Japanese gastric cancer patients, and it was created with a Cox regression model. Fifteen clinical variables, which were obtained at pretreatment, were collected and registered. Data of two independent cohorts of patients from Seoul St. Mary's Hospital (1001 patients), and the University of Verona (389 patients) formed the external validation cohorts. The model was validated internally and externally using measures of discrimination (Harrell's C‐index), calibration, and decision curve analysis.

**Results:**

The developed nomogram showed good discrimination, with a C‐index of 0.855; that of the American Joint Committee on Cancer (AJCC) clinical stage was 0.819. In the external validation procedure, the C‐indexes were 0.856 (AJCC, 0.795) in the Seoul St. Mary's cohort and 0.714 (AJCC, 0.648) in the University of Verona cohort. The nomogram performed well in the calibration and decision curve analyses when applied to both the internal and external validation cohorts. A stage‐specific subset survival analysis of the three risk groups calculated using the nomogram also showed the superiority of nomogram‐prediction when compared to AJCC.

**Conclusion:**

This new pretreatment model accurately predicts OS in gastric cancer and can be used for patient counseling in clinical practice and stratification in clinical trials.

## Graphical abstract

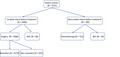

## 1 INTRODUCTION

To choose appropriate therapy and provide an estimation of prognosis, the American Joint Committee on Cancer (AJCC) has defined gastric cancer staging.^1^ AJCC staging is composed of three variables including tumor depth (T), nodal metastasis (N), and distant metastasis (M). However, other promising prognosticators, have been reported.^2^, ^3^, ^4^

Nomograms have been developed to quantify risk by combining several prognostic factors in some malignancies.^5^, ^6^ We first developed a gastric cancer nomogram based on a Western database,^7^ followed by additional nomograms based on Asian databases.^8^, ^9^ All studies emphasizing nomograms have been more informative than those using AJCC staging. However, all previous gastric cancer nomograms were constructed using pathologic variables obtained after gastrectomy.

Selection of a treatment strategy is primarily based on the pretreatment clinical stage (cStage). Guidelines in Japan,^10^ the United States,^11^ and Europe^12^ state that the treatment strategy should be determined based on cStage. Furthermore, an increasing number of patients are undergoing neoadjuvant chemotherapy (NAC) in Western countries; therefore, the need for accurate assessment of cStage is vital. Studies demonstrating a significant correlation between cStage and survival have been reported recently.^13^, ^14^

The AJCC has increasingly recognized the need for more personalized probabilistic predictions than those delivered by ordinal systems, particularly through the use of accurate risk assessment models.^15^ Despite this situation, there have been no published pretreatment gastric cancer nomograms constructed using pretreatment variables.

We therefore sought to develop the first pretreatment gastric cancer nomogram including the patients receiving nonsurgical therapy, with external validation in independent cohorts, to assist ongoing efforts toward a more personalized paradigm according to the AJCC Precision Medicine Core.

## 2 MATERIALS AND METHODS

### 2.1 Patients in developing cohort and pretreatment prognostic variables

Data registered in Shizuoka Cancer Center database were used in developing a nomogram. We enrolled 5231 patients with primary gastric cancer between October 2002 and July 2017.

We collected pretreatment information on patient demographics (age, sex, Eastern Cooperative Oncology Group Performance Status (ECOGPS)), and tumor characteristics (location, size, clinical depth (cT), number of positive regional nodes on CT (cN‐Number), location of positive nodes on CT (cN‐Location), liver metastasis, peritoneal dissemination, other sites of distant metastasis (cM), macroscopic type, histology (via endoscopic biopsy), serum carcinoembryonic antigen (CEA), and serum carbohydrate antigen 19‐9 (CA19‐9)) (see Supporting Methods and Supporting Table 1).

This study was approved by the Institutional Review Board of Shizuoka Cancer Center (T29‐34‐29‐1, T30‐4‐30‐1).

### 2.2 Patients in external validation cohort

Two independent cohorts, including 1001 patients from Seoul St. Mary's Hospital (Seoul, Republic of Korea) and 389 patients from the University of Verona (Verona, Italy), formed external validation cohorts. These external validation cohorts of this study were approved by the Institutional Review Board of Seoul St. Mary's Hospital (KC17RESI0281) and the Italian Research Group for Gastric Cancer (GIRCG).

### 2.3 Statistical methods

Continuous variables were modeled as restricted cubic splines for potential nonlinear effect. Before modeling, we adopted log‐transformed CEA and CA19‐9 as predictors to reduce the skewness of the data. The endpoint in the nomogram was overall survival (OS). The nomogram was created with a Cox proportional hazard model, and it was validated internally and externally using measures of discrimination (Harrell's C‐index), calibration, and decision curve analysis (see Supporting Statistical Methods). R version 3.4.4 (The R Foundation for Statistical Computing, Vienna, Austria) was used to perform all analyses.

## 3 RESULTS

### 3.1 Patient demography and treatment

**FIGURE 1:**
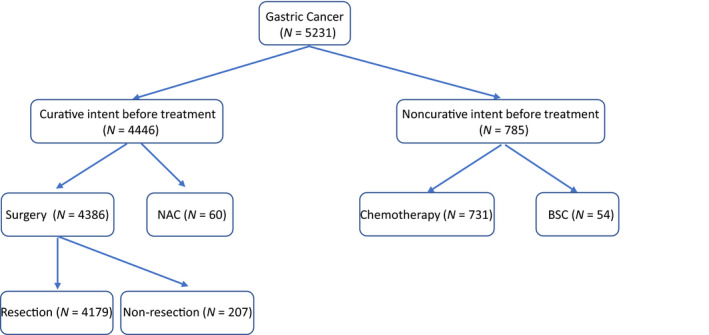
Summary of treatment performed after diagnosis. Abbreviations: BSC, best supportive care; NAC, neoadjuvant chemotherapy

Descriptive statistics appear in Table 1. The therapeutic strategy was determined by the treatment guideline or protocol of participating clinical trials based on pretreatment tumor progression and patient condition. Treatments performed among study participants are summarized in Figure 1.

**TABLE 1:** Patient characteristics and pretreatment clinical variables

Pretreatment variables		
Location		
Lower (Antrum, Pylorus)	1466	(28.0)
Middle (Body)	2182	(41.7)
Upper (Cardia, Fundus)	1034	(19.8)
Entire (Overlapping Stomach)	432	(8.3)
EGJ (Esophagogastric junction tumor)	117	(2.2)
Tumor Size (mm)		
Minimum	2	
First quartile	28	
Median	40	
Mean	52	
Third quartile	70	
Maximum	250	
cT		
cT1a = Mucosa	1101	(21.0)
cT1b = Submucosa	1227	(23.5)
cT2 = Propria musclaris	532	(10.2)
cT3 = Subserosa	345	(6.6)
cT4a = Serosal invasion	1809	(34.6)
cT4b = Adjacent organ involvement	217	(4.1)
cN (Number)		
Minimum	0	
First quartile	0	
Median	0	
Mean	2.1	
Third quartile	3	
Maximum	42	
cN (Location)		
cN0	3398	(65.0)
cN1 (Perigastric or No. 110 if EGJ)	922	(17.6)
cN2a (No. 7, 8a, 9)	377	(7.2)
cN2b (No 10, 11p, 11d, 12a or 19, 20, 111 if EGJ)	95	(1.8)
cNM (Nonregional LN, intra‐abdominal)	439	(8.4)
Liver metastasis		
Negative	4889	(93.5)
Solitary	41	(0.8)
Multiple	301	(5.8)
Peritoneum		
Negative	4861	(92.9)
Positive	370	(7.1)
cM		
Negative	5103	(97.6)
Positive	128	(2.4)
Macroscopic type		
Type 0	2670	(51.0)
Type 1	169	(3.2)
Type 2	830	(15.9)
Type 3	1088	(20.8)
Type 4	474	(9.1)
Histology		
G1 (Well‐differentiated type)	877	(16.8)
G2 (Moderately differentiated type)	1226	(23.4)
G3 (Poorly differentiated or Undifferentiated type)	3128	(59.8)
Age		
Minimum	19	
First quartile	60	
Median	67	
Mean	66	
Third quartile	74	
Maximum	95	
Sex		
Female	1644	(31.4)
Male	3587	(68.6)
ECOG Performance Status (PS)		
0	4360	(83.3)
1	611	(11.7)
2	198	(3.8)
3 or 4	62	(1.2)
Serum CEA (ng/mL)		
Minimum	0.5	
First quartile	1.4	
Median	2.3	
Mean	46.8	
Third quartile	4.0	
Maximum	38 640.0	
Serum CA19‐9 (U/mL)		
Minimum	2	
First quartile	3	
Median	8	
Mean	637	
Third quartile	18	
Maximum	708 200	

Note

cM excludes liver, peritoneum, intra‐abdominal nonregional metastasis.

### 3.2 Survival outcomes

**FIGURE 2:**
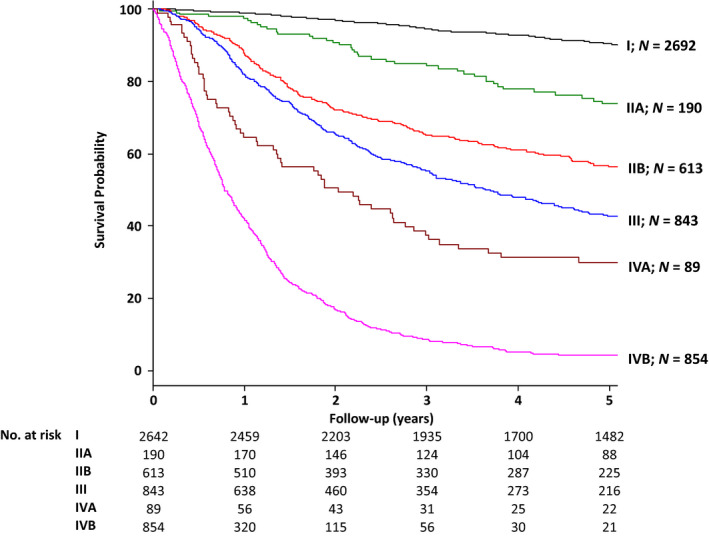
Kaplan‐Meier survival curves of overall survival by American Joint Committee on Cancer stage (AJCC) grouping in developing cohort, with numbers at risk

Figure 2 shows OS curves according to AJCC cStage. From the step‐down model reduction method, all 15 variables were selected to construct the nomogram for OS (Table 2). This nomogram can be used to predict probability of patient death due to any cause at 1, 3, and 5 years

. The equation predicting 5‐year OS is presented (see Supporting Result).

**TABLE 2:** Multivariable Cox proportional hazards model of pretreatment variables for overall survival

Pretreatment variables	Chi‐Square	*P*	HR	95% CI
Location	11.62	.020		
Lower			1.18	1.04~1.34
Middle			1	
Upper			1.20	1.04~1.37
Entire			1.24	1.03~1.50
EGJ			1.31	1.03~1.66
Tumor size (mm)	1.79	.181		
			1.06	0.97~1.16
cT	79.20	<.001		
cT1a			0.24	0.17~0.34
cT1b			0.30	0.21~0.42
cT2			0.43	0.33~0.55
cT3			0.62	0.51~0.76
cT4a			1	
cT4b			1.13	0.96~1.33
cN (Number)	19.15	<.001	1.08	
				1.04~1.12
cN (Location)	38.81	<.001		
cN0			1	
cN1			1.20	1.05~1.38
cN2a			1.28	1.07~1.53
cN2b			1.96	1.47~2.57
cNM			1.82	1.48~2.24
Liver Metastasis	73.04	<.001		
Negative			1	
Solitary			1.69	1.18~2.42
Multiple			2.02	1.71~2.39
Peritoneum	83.82	<.001		
Negative			1	
Positive			1.98	1.71~2.30
cM	6.07	.014		
Negative			1	
Positive			1.30	1.05~1.60
Macroscopic type	82.43	<.001		
Type 0			1	
Type 1			1.62	1.16~2.27
Type 2			1.01	0.75~1.36
Type 3			1.30	0.97~1.73
Type 4			2.30	1.67~3.16
Histology	24.94	<.001		
G1			0.73	0.62~0.86
G2			0.79	0.70~0.88
G3			1	
Age	44.79	<.001		
			1.50	1.40~1.61
Sex	1.08	.299		
Female			0.95	0.85~1.05
Male			1	
ECOG PS	86.92	<.001		
0			1	
1			1.45	1.27~1.66
2			2.10	1.74~2.53
3 or 4			2.44	1.82~3.26
Serum CEA (ng/mL)	6.10	.014		
			1.15	1.07~1.25
Serum CA19‐9 (U/mL)	5.72	.017		
			1.06	1.01~1.10

Abbreviations: CI, confidence interval; HR, hazard ratio.

**FIGURE 3:**
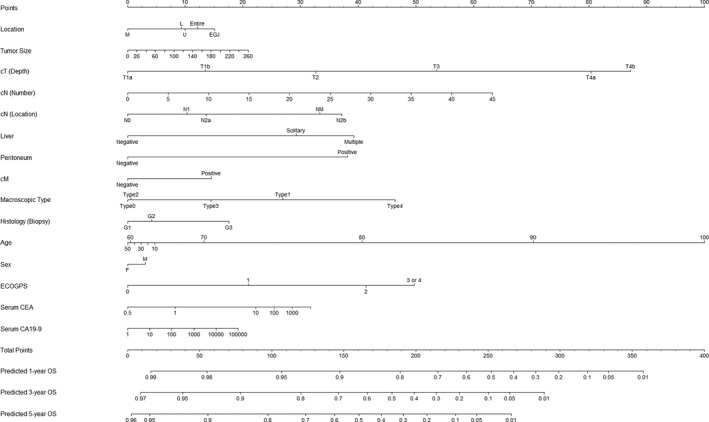
Pretreatment gastric cancer nomogram. This nomogram provides a method to calculate 1‐, 3‐, and 5‐y probability of survival. Add the scores for each covariate together and locate the total score on the total points axis. Draw a line straight down to the 1‐, 3‐, and 5‐y OS axes to obtain the probability. Abbreviations: CA19‐9, carbohydrate antigen 19‐9; CEA, carcinoembryonic antigen; cM, distant metastasis except metastasis in intra‐abdominal nonregional lymph node, liver metastasis, and peritoneal dissemination; ECOGPS, Eastern Cooperative Oncology Group performance status; Liver, liver metastasis; ln, natural logarithm; OS, overall survival; Peritoneum, peritoneal dissemination.

### 3.3 Evaluation of nomogram in the developing cohort

**FIGURE 4:**
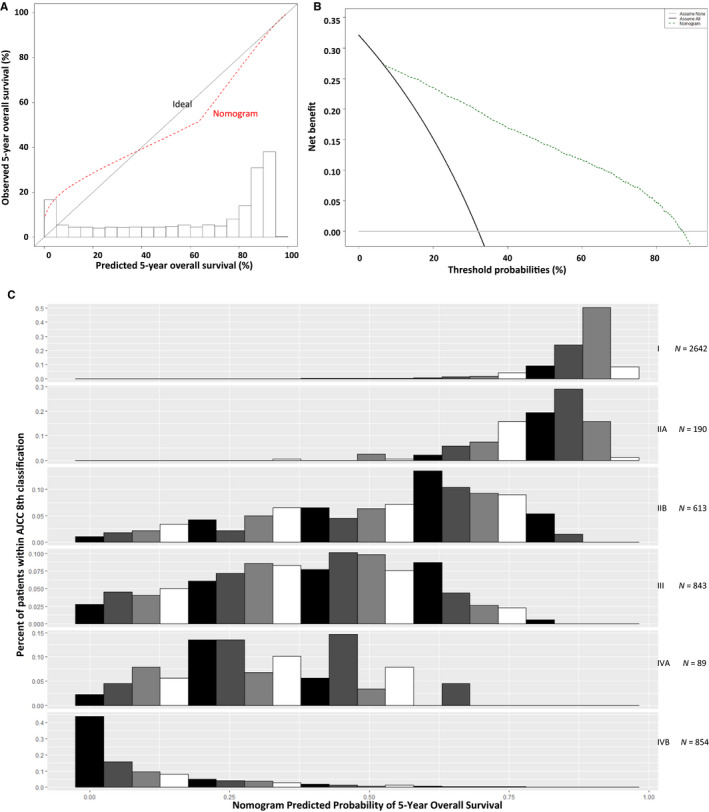
Discriminatory performance of the nomogram. Data are from the development cohort. A, Calibration plot of the overall survival nomogram. B, Decision curve to plot the net benefit achieved by making clinical decisions based on the final multivariable model prediction at 5 y, for overall survival. C, Distribution of nomogram predictions within each American Joint Committee on Cancer (AJCC) stage grouping, for overall survival

The C‐index for the model was 0.855 (95% CI, 0.848‐0.863); that of the AJCC was 0.819 (95% CI, 0.810‐0.828). The calibration appeared to be accurate for predicting 5‐year OS (Figure 4A). Figure 4B shows the results of decision curve analysis. Compared with scenarios where no prediction model was used for a pretreatment decision (ie, treat all or treat none), the nomogram had a favorable net benefit across a wide range of decision threshold probabilities, between approximately 15% and 90% OS at 5 years. Note the heterogeneity in nomogram‐predicted probability present within AJCC stages (Figure 4C).

### 3.4 Evaluation of nomogram in the external validation cohort

The OS curve of the Seoul St. Mary's cohort according to the latest AJCC cStage and the OS curve of the Verona cohort according to AJCC cStage are presented (see Supporting Figure 1A, B). In external validation of the Seoul St. Mary's cohort, the C‐index applied to the nomogram was 0.856 (95% CI, 0.823‐0.879), compared with 0.795 (95% CI, 0.776‐0.814) when applied to the AJCC. In external validation of the Verona cohort, the C‐index applied to the nomogram was 0.714 (95% CI, 0.681‐0.746), compared with 0.648 (95% CI, 0.629‐0.667) applied to the AJCC. The predicted and observed outcomes were in good agreement (see Supporting Figure 2A, B). This pretreatment nomogram also yielded a wide range of clinical net benefit in external cohorts (see Supporting Figure 3A, B).

### 3.5 Additional analysis

In addition, a stage‐specific subset survival analysis of the three risk groups calculated using the nomogram was performed. The definition of three risk group were below: low‐risk group, nomogram‐predicted 5‐year survival rate (5‐years) is 70‐100%, intermediate‐risk group, nomogram‐predicted 5‐years is 30‐70%, and high‐risk group, nomogram‐predicted 5‐years is 0‐30%. The rationale for using three risk groups and their cutoff values is based on survival outcomes stratified AJCC clinical staging that decides therapeutic planning for gastric cancer (see Supporting Table 2).

**FIGURE 5:**
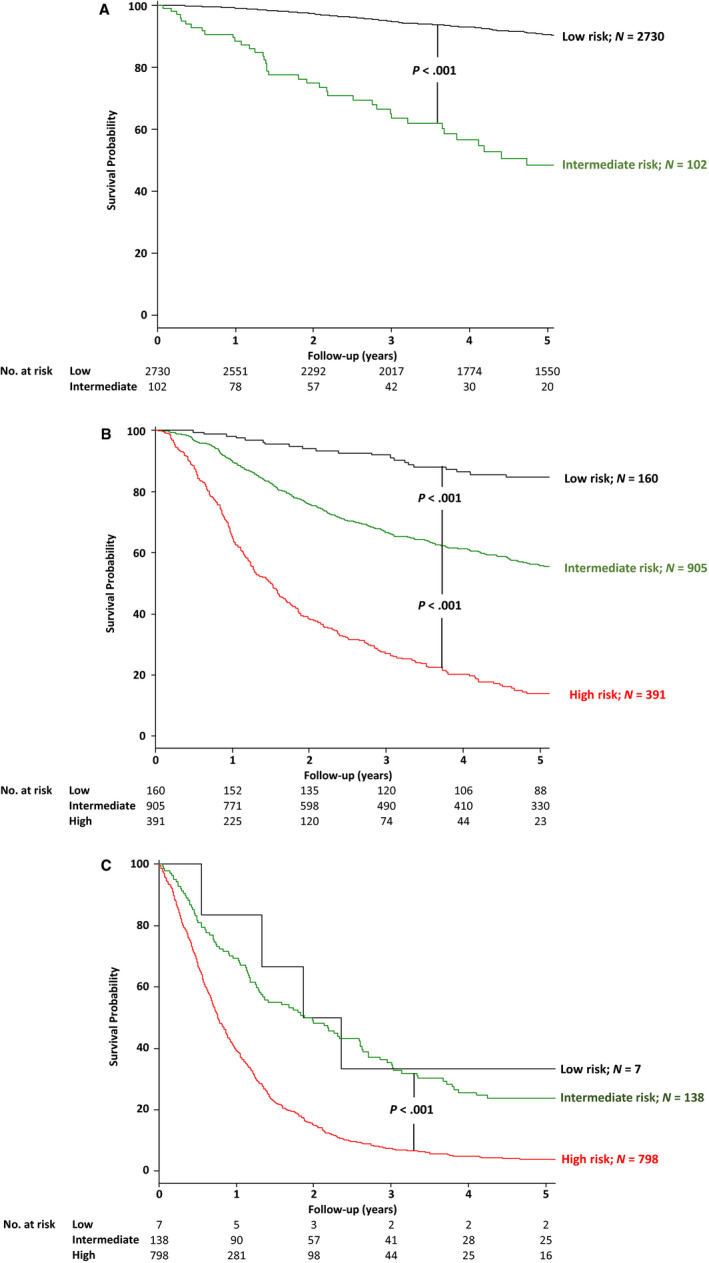
Kaplan‐Meier survival curves of the overall survival of patients stratified into three risk groups according to nomogram‐predicted 5‐y survival rate in the development cohort, with the numbers at risk. A, cStage I/ IIA, B, cStage IIB/III, and C, cStage IVA/IVB

The intermediate‐risk group had a significantly poorer survival than the low‐risk group in cStage I/IIA (*P* < .001) and cStage IIB/III (*P* < .001) (Figure 5A,B). The high‐risk group had a significantly poorer survival than the intermediate‐risk group in cStage IIB/III (*P* < .001) and cStage IVA/IVB (*P* < .001) (Figure 5B,C). There may be a possibility that 102 patients in cStage I/IIA (3.6%) and 391 (26.9%) patients in cStage IIB/III have been undertreated, and 160 patients (11.0%) in cStage IIB/III have been unnecessarily overtreated. In addition, the AJCC staging system lost its predictive ability in each nomogram‐predicted risk group (see Supporting Figure 4A‐C), except for cStage IVB. We also performed these analyses on external validation cohorts. Clear survival distributions were found in the same AJCC stage in both cohorts (see Supporting Figures 5A, B and 6A‐C). In the reverse analysis in both external cohorts, the AJCC staging system lost its predictive ability in each risk group (see Supporting Figures 7A‐C and 8A‐C).

## 4 DISCUSSION

To our best knowledge, this is the first study to build a gastric cancer nomogram using only pretreatment clinical variables and including not only patients receiving initial surgery, but also those receiving NAC, chemotherapy, or BSC. Part of our data,^16^ as well as that of the National Cancer Data Base (NCDB) in the United States,^14^ was used for constructing the cStage of the latest edition of the AJCC.^1^

This study has several strengths. First, new nomogram was successfully validated internally and externally in two independent cohorts, not only with the C‐index or calibration plots but also decision curve analysis. Decision curve analyses assess the clinical usefulness of prediction tools by quantifying the net benefits when different threshold probabilities are considered. Thresholds are attractive for use in clinical trials. In internal validation, for example, if the inclusion criteria for a neoadjuvant clinical trial is more than 30% the risk for OS at 5 years, a nomogram‐based decision would have a net benefit of approximately 0.17, which is 0.17 greater than assuming all patients need treatment. In other words, use of the nomogram would lead to the equivalent of 17% reduction in unnecessary treatment without a decrease in the number of patients who duly need treatment.

Second, our study showed the clear survival distributions according to the nomogram‐based risk groups in the stage‐specific evaluation both in developing and external cohorts. In addition, the AJCC staging system lost its predictive ability in each nomogram‐predicted risk group. These results indicate that some patients of the intermediate‐risk group (cStage IIB/III) are actually at a high or low risk, and some patients in the low‐risk group (cStage I/IIA) are actually at an intermediate risk. We are convinced that the use of the nomogram prediction could aid in avoiding undertreatment or unnecessary overtreatment for a large number of patients in routine practice. For example, NAC is the standard treatment for an advanced gastric cancer in Western countries such as Italy. Even for a cStage I cancer, patients in the intermediate‐ or high‐risk groups become candidates for NAC according to the nomogram prediction. Conversely, for cStage III cancer, the low‐risk group could avoid NAC. A variety of treatments can be planned for the patients in stage IV of the disease. These will greatly depend on the patient's condition and the metastasis status. Therefore, it is difficult to assess if the performed treatment was accurate, or an overtreatment was performed. Nonetheless, the patients in the high‐risk group had significantly poorer outcomes than those in the intermediate‐risk group. Therefore, the nomogram may be useful for treatment selection even in cStageIV. In addition, we believe that nomogram prediction plays a vital role not only in the choice of accurate therapy in practice, but also in patient recruitment/enrichment for prospective clinical trials. For patients with cStage III gastric cancer, the standard therapy was initial surgery in Japan and Korea. In recent years, however, a clinical trial of NAC is ongoing in both countries. Using nomograms to define eligibility is expected to allow truly intermediate/high‐risk patients to enter the trial, while excluding patients who are not of sufficient risk relative to high‐risk patients in the traditional sense. Weiser et al. suggested that nomograms would considerably reduce the sample size requirement, which could reduce operation time and cost.^17^

Third, this nomogram was constructed using pretreatment factors, which are recommended to collect and register by the AJCC and Union for International Cancer Control (UICC). For establishing a statistical risk assessment model as a future staging system, the AJCC presents the recommended pretreatment variables in the Cancer Staging Manual,^1^ such as tumor location, tumor size, location of clinical positive nodes, sites of distant metastasis, serum CEA, and serum CA19‐9, in parallel with traditional factors including depth of invasion (cT) and number of clinically malignant nodes (cN). The eighth UICC TNM classification includes age as a host‐related prognostic factor.^18^ We also included sex, histology, macroscopic type, and ECOG‐PS.

Inclusion of 15 variables seems complex; however, these variables can be collected easily, even in general hospitals. Recent advancements in computer technologies provide easy ways to use online software. With use of online calculator, some cumbersome problems could be solved. Some prediction models are open access on the Cleveland Clinic website (http://riskcalc.org/).^19^

Previous studies have reported better survival outcomes in Asian patients than Western patients.^20^, ^21^ Consistent with these findings, our results for calibration plots in external validation of the Verona (Italy) cohort were favorable. Recent studies report that survival does not differ significantly between Asian and Western patients when comparing similar subtypes of gastric cancer^22^ or when adjusting by Han's Korean nomogram.^23^ These studies speculate that gastric cancer is a heterogeneous disease and when adjusted by many variables, the difference in survival outcome between Asian and Western patients may disappear.

The present study has several limitations. Although we constructed a novel risk model, its predictive accuracy can be improved. Another limitation is that the two external validation cohorts were based on surgical databases. The St. Mary's cohort included patients with potentially good outcomes because they were treated with preoperative curative intent followed by curative gastrectomy. Even in cStage I/II/III patients treated with curative intent, quite a few patients had incurable factors.^13^ The Verona cohort included some cStageIV patients; however, surgery was also assumed. One feature of our nomogram is that it can predict the treatment outcomes of nonsurgical as well as resected cases; however, these variations might cause some biases.

In conclusion, the newly developed gastric cancer pretreatment nomogram will be useful for planning clinical trial designs, selecting appropriate therapy, and for patient counseling in routine practice. This model will improve on the traditional AJCC system in the future.

## 5 ETHICAL APPROVAL STATEMENT

The study protocol has been approved by the Institutional Review Board of all participating institutes. The ethical guidelines of the World Medical Association Declaration of Helsinki—Ethical Principles for Medical Research Involving Human Subjects were fully conformed when conducting the present study.

## CONFLICT OF INTEREST DISCLOSURES

The authors made no disclosures.

## AUTHOR CONTRIBUTIONS

**Etsuro Bando:** Conceptualization, formal analysis, investigation, methodology, and writing—original draft. **Xinge Ji:** Formal analysis, methodology, and writing—review and editing. **Michael W. Kattan:** Supervision, project administration and writing—review and editing. **Ho Seok Seo:** Data curation and writing—review and editing. **Kyo Young Song:** Resources and writing—review and editing. **Cho‐Hyun Park:** Resources and writing—review and editing. **Maria Bencivenga:** Data curation and writing—review and editing. **Giovanni de Manzoni:** Resources and writing—review and editing. **Masanori Terashima:** Project administration, and writing—original draft. **Precis for use in the Table of Contents:** The newly developed gastric cancer pretreatment nomogram can accurately predict overall survival and thereby improve risk stratification and patient selection for routine clinical decision‐making and future clinical trials. This model will improve on the traditional categorical AJCC system in the future.

## Supporting information

DataS1

## Data Availability

The study data of development cohort are accessible.
