# Supplementary material for: Development and validation of a pretreatment nomogram to predict overall survival in gastric cancer
Source: Cancer Med. 2020 Jun 26;9(16):5708–18. doi: 10.1002/cam4.3225 (PMC7433838; doi:10.1002/cam4.3225)

**Supporting Information**

SUPPORTING METHODS……………………………………………………….……………2

SUPPORTING STATISTICAL METHODS……………………………………….…………..4

REFERENCES.………………………………………………………………………………....6

SUPPORTING RESULT…………………………………………………….…………………7

SUPPORTING TABLES……………………………………………………………………….8

Supporting Table 1. The definition of categories of pretreatment variables………….……...8

Supporting Table 2. The definition of nomogram-predicted three risk grouping…………....9

SUPPORTING FIGURES……………………………………………………………………...10

Supporting Figure 1. Kaplan–Meier survival curves of overall survival in external validation cohorts…………………………………………...………………………………………….10

Supporting Figure 2. Calibration plots for external validation…………………….………...11

Supporting Figure 3. Decision curves to plot the net beneﬁt in the external validation cohorts.12

Supporting Figure 4. Kaplan–Meier survival curves of overall survival by AJCC stage grouping in the development cohort…………………………………….….……………………..…...13

Supporting Figure 5. Kaplan-Meier survival curves of the overall survival of the patients stratified into three risk groups according to the nomogram-predicted 5-year survival rate in the Seoul St. Mary Hospital (Seoul, Republic of Korea) external validation cohort………….…15

Supporting Figure 6. Kaplan-Meier survival curves of the overall survival of the patients stratified into three risk groups according to the nomogram-predicted 5-year survival rate in the Verona University (Verona, Italy) external validation cohort………………………………16

Supporting Figure 7. Kaplan–Meier survival curves of overall survival by AJCC stage grouping in the Seoul St. Mary Hospital (Seoul, Republic of Korea) external validation cohort……...18

Supporting Figure 8. Kaplan–Meier survival curves of overall survival by AJCC stage grouping in the Verona University (Verona, Italy) external validation cohort………………...………20

SUPPORTING METHODS

The definition of categories of pretreatment variables are summarized in Supporting Table1.

Eastern Cooperative Oncology Group Performance Status (ECOGPS)^1^ was described according to four score categories; 0, 1, 2, and “3 or 4”. The definition of ECOGPS was as follows: 0; fully active, able to carry on all pre-disease performance without restriction, 1; restricted in physically strenuous activity but ambulatory and able to carry out work of a light or sedentary nature, 2; ambulatory and capable of all selfcare but unable to carry out any work activities, up and about more than 50% of waking hours, 3; capable of only limited selfcare, confined to bed or chair more than 50% of waking hours, 4; completely disabled, cannot carry on any selfcare, totally confined to bed or chair. Tumor location was subdivided into five categories: L; antrum, M; body, U; cardia, Entire; overlapping lesion, EGJ; esophagogastric junction tumor. Tumor length was measured by endoscopy or barium imaging.

Diagnosis of clinical tumor depth (cT) and clinically positive lymph nodes (cN) was determined according to a previously published report.^2^ cN-Number was treated as continuous variables, not converted to the current TNM category (N0/1/2/3a/3b). cN-Location was classified according to the following categories: N0; no positive nodes, N1; positive nodes in the perigastric area (No. 1, 2, 3a, 3b, 4sa, 4sb, 4d, 5 and 6, or No. 110 (paraesophageal nodes) if EGJ tumor), N2a; positive nodes in the suprapancreatic area (No. 7, 8a, and 9), N2b; positive nodes in the splenic hilum area, along the splenic artery, and in the hepatoduodenal ligament (No. 10, 11p, 11d, and 12a, or No. 19 (infradiaphragmatic nodes), No. 20 (esophageal hiatus), and No. 111 (supradiaphragmatic nodes) if EGJ tumor), and NM; positive nodes in intra-abdominal nonregional nodes (No. 12b/p, 13, 14a/v, 15, 16a/b, 17, and 18). This classification is referred to in the second English Edition of the Japanese Classification of Gastric Carcinoma and Japanese gastric cancer treatment guidelines.^3,4^ For cN2 classification only, we used our own classification. cN2a is the node within the area of limited D1+ lymphadenectomy, while cN2b is the node which does not dissected unless radical D2 dissection was performed.

Liver and peritoneum metastases were analyzed separately from cM due to their high frequency. We divided positive liver metastasis into solitary and multiple because solitary metastasis might be curable by liver resection.^5^ cM included metastasis to the lung, pleura, bone, central nervous system, skin, muscle, breast, spleen, adrenal glands, and extra-abdominal nodes. Macroscopic type was classified into Type 0 to Type 4 according to the Borrmann classification. Histology was classified into three groups: G1 (well differentiated), G2 (moderately differentiated), and G3 (poorly differentiated or undifferentiated).

**SUPPORTING STATISTICAL METHODS**

Cox proportional hazards model was performed to develop the nomogram using all potential variables and was reduced to find the best-fitting parsimonious model. The model reduction process identifies the variable that has the smallest reduction in R2 and then removes it from the model;^6^ this process is continued until all variables are removed from the model. At each removal, the C-index is calculated, and the process is stopped when the change of the C-index is less than 0.001.

The models’ performance was first measured internally. Internal performance was measured according to discrimination and calibration. Discrimination was measured with the C-index, which is the area under the receiver operating curve (ROC) and describes the model’s ability to distinguish between a patient at higher risk of event occurrence from a patient who is at lower risk. The C-index ranges from 0.5 to 1, where 1 indicates that the model has perfect discrimination and 0.5 indicates that the model does no better than chance.^7^ In addition to the Harrell’s C-index, we performed a stage-specific subset survival analysis based on the newly defined risk groups to determine whether the nomogram had better predictive ability than the AJCC staging system.

Calibration was assessed using a plot that measures the relationship between the model’s predicted risk and the actual fraction of patients with the outcome. A straight 45° line indicates perfect calibration; any deviation above or below the 45° line indicates under- and overprediction, respectively. All measurements of performance in internal validation procedure were validated using a 200-bootstrap resampling process to correct for the bias of overfitting. Clinical benefit-centered accuracy of the final model was evaluated using decision curve analysis,^8^ to identify the range of threshold probabilities in which the risk score has higher net benefit than “assume all” and “assume none”.

External validation was performed by comparing the predicted 5-year OS probabilities of patients in two independent external validation cohorts, and the observed actual 5-year OS probabilities of the same patients. The external validation procedures were also performed using the C-index, calibration curves, and decision curve analysis.

**REFERENCES**

1. ECOG-ACRIN Cancer Research Group: ECOG Performance Status. <http://ecog-acrin.org/resources/ecog-performance-status>
2. Bando E, Makuuchi R, Tokunaga M, Tanizawa Y, Kawamura T, Terashima M. Impact of clinical tumor-node-metastasis staging on survival in gastric carcinoma patients receiving surgery. Gastric Cancer. 2017; 20: 448-456.
3. Japanese Gastric Cancer Association: Japanese Classification of Gastric Carcinoma (English ed 2). Gastric Cancer. 1998: 1: 10-24.
4. Japanese Gastric Cancer Association: Japanese gastric cancer treatment guidelines 2014 (ver 4). Gastric Cancer. 2017; 20: 1-19.
5. Markar SR, Mikhail S, Malietzis G, et al. Influence of Surgical Resection of Hepatic Metastases From Gastric Adenocarcinoma on Long-term Survival: Systematic Review and Pooled Analysis. Ann Surg. 2016; 263: 1092-1101.
6. Harrell FE: Regression Modeling Strategies – With Applications to Linear Models, Logistic Regression, and Survival Analysis (ed 2). New York, Springer, 2015.
7. Harrell FE Jr, Lee KL, Mark DB. Multivariable prognostic models: Issues in developing models, evaluating assumptions and adequacy, and measuring and reducing errors. Stat Med. 1996; 15: 361-387.
8. Vickers AJ, Elkin EB. Decision curve analysis: A novel method for evaluating prediction models. Med Decis Making. 2006; 26: 565-574.

**SUPPORTING RESULT**

Base is 0.6879258 (SerumCEA.log = 0.83290912, SerumCA19-9.log = 2.0794415)

{-1.8425423

+ 0.010846172 * (Location = "U") - 0.16749 * (Location = "M")

+ 0.050019648 * (Location = "Entire") + 0.10269293 * (Location = "EGJ")

+ 0.0014430737 * Tumor Size

+ 0.24252436 * (cT = "T1b") + 0.58495043 * (cT = "T2")

+ 0.96168736 * (cT = "T3") + 1.4407734 * (cT = "T4a")

+ 1.5651553 * (cT = "T4b")

+ 0.025207497 * cN (Number)

+ 0.18445617 * (cN (Location) = "N1") + 0.24524056 * (cN (Location) = "N2a")

+ 0.66597235 * (cN (Location) = "N2b") + 0.59722426 * (cN (Location) = "NM")

+ 0.5244548 * (Liver = "Solitary") + 0.70337606 * (Liver = "Multiple")

+ 0.68491897 * (Peritoneum = "Positive") + 0.26087311 * (cM = "Positive")

+ 0.48301951 * (Macroscopic Type = "Type1")

+ 0.0099029446 * (Macroscopic Type = "Type2")

+ 0.25913262 * (Macroscopic Type = "Type3")

+ 0.83252198 * (Macroscopic Type = "Type4")

+ 0.07494354 * (Histology = "G2") + 0.31581898 * (Histology = "G3")

- 0.002108175 * Age + 4.1157608e-05 * max(Age - 51, 0)**3

- 9.6034419e-05 * max(Age - 67, 0)**3 + 5.4876811e-05 * max(Age - 79, 0)**3

+ 0.05512007 * (Sex = "M")

+ 0.37494832 * (ECOGPS = "1") + 0.74113905 * (ECOGPS = "2")

+ 0.89155126 * (ECOGPS = "3 or 4")

+ 0.21417678 * SerumCEA.log

- 0.027607203 * max(SerumCEA.log + 0.10536052, 0)**3

+ 0.044884683 * max(SerumCEA.log - 0.83290912, 0)**3

- 0.017277481 * max(SerumCEA.log - 2.3321439, 0)**3

+ 0.029899589 * SerumCA19-9.log;

| **SUPPORTING TABLES**  **Supporting Table 1**. The definition of categories of pretreatment variables. | | | | | | |
| --- | --- | --- | --- | --- | --- | --- |
| **Pretreatment variables** |  |  |  |  |  |  |
| **Categorical variables** |  |  |  |  |  |  |
| Location | L | U | M | Entire | EGJ |  |
| cT (Depth) | T1a | T1b | T2 | T3 | T4a | T4b |
| cN (Location) | N0 | N1 | N2a | N2b | NM |  |
| Liver | Negative | Solitary | Multiple |  |  |  |
| Peritoneum | Negative | Positive |  |  |  |  |
| cM (Distant metastasis) | Negative | Positive |  |  |  |  |
| Macroscopic Type | Type0 | Type1 | Type2 | Type3 | Type4 |  |
| Sex | F | M |  |  |  |  |
| ECOGPS | 0 | 1 | 2 | 3 or 4 |  |  |
| Histology (Biopsy) | G1 | G2 | G3 |  |  |  |
| **Continuous variables** |  |  |  |  |  |  |
| Tumor Size (mm) |  |  |  |  |  |  |
| cN (Number) |  |  |  |  |  |  |
| Age |  |  |  |  |  |  |
| Serum CEA (ng/mL) |  |  |  |  |  |  |
| Serum CEA19-9 (U/mL) |  |  |  |  |  |  |
| Liver, liver metastasis; Peritoneum, peritoneal dissemination; cM, distant metastasis except metastasis in intra-abdominal nonregional lymph node, liver metastasis, and peritoneal dissemination; ECOGPS, Eastern Cooperative Oncology Group performance status; CEA, carcinoembryonic antigen; CA19-9, carbohydrate antigen 19-9. | | | | | | |

| **Supporting Table 2.** The definition of nomogram-predicted three risk grouping based on relationship between the recommended treatment per Japanese/U.S. guidelines and the AJCC clinical staging. | | | | | |
| --- | --- | --- | --- | --- | --- |
|  | **Japanese guideline** | | **U.S. guideline** | **5-year survival rate** | **Risk category** |
| **AJCC** | **Practice** | **Trial** | **Practice** | **predicted by AJCC** |  |
| cStageI | Surgery | Surgery | Surgery (T1N0) | 70-100% | Low |
|  |  |  | Surgery, NAC, Neo-CRT (T2N0) |  |  |
| cStageIIA | Surgery | Surgery | Surgery (T1N+) |  |  |
|  |  |  | Surgery, NAC, Neo-CRT (T2N+) |  |  |
| cStageIIB | Surgery | Surgery, NAC | Surgery, NAC, Neo-CRT | 30-70% | Intermediate |
| cStageIII | Surgery, NAC | Surgery, NAC | Surgery, NAC, Neo-CRT |  |  |
| cStageIVA | Surgery, Chemotherapy, CRT, NAC, Neo-CRT, Conversion, BSC | | | 0-30% | High |
| cStageIVB |  |  |  |  |  |
| NAC, neoadjuvant chemotherapy; CRT, chemoradiation therapy; Neo-CRT, CRT before surgery; Conversion, conversion surgery;  BSC, best supportive care; AJCC, American Joint Committee on Cancer | | | | | |

**SUPPORTING FIGURES**

**Supporting Figure 1.** Kaplan–Meier survival curves of overall survival by American Joint Committee on Cancer (AJCC) stage grouping, with numbers at risk.

(A) cohort of Seoul St. Mary’s Hospital (Seoul, Republic of Korea), and (B) cohort of University of Verona (Verona, Italy)


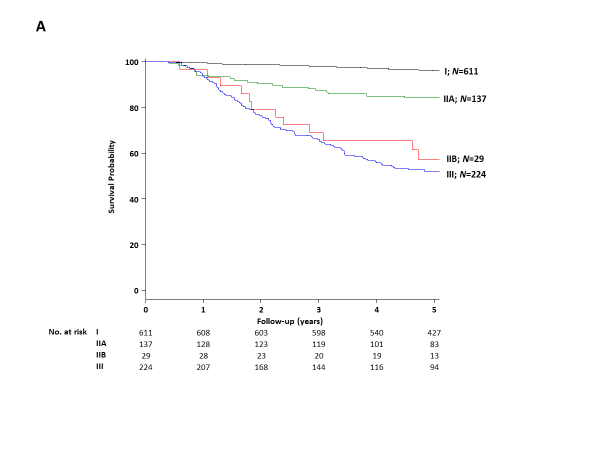


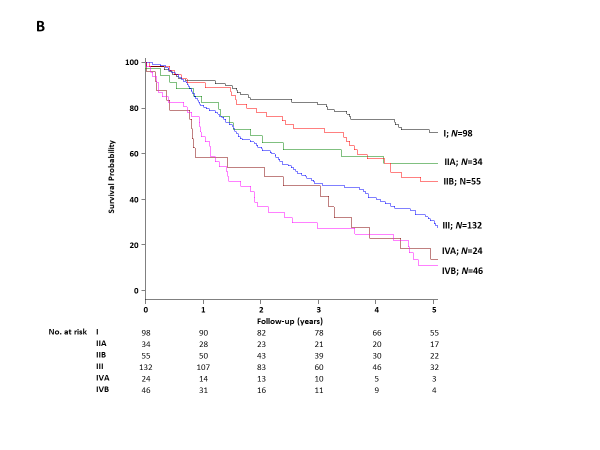


**Supporting Figure 2.** Calibration plots for external validation of the overall survival nomogram.

1. Seoul St. Mary’s Hospital validation cohort (Seoul, Republic of Korea) and (B) University of Verona validation cohort (Verona, Italy).


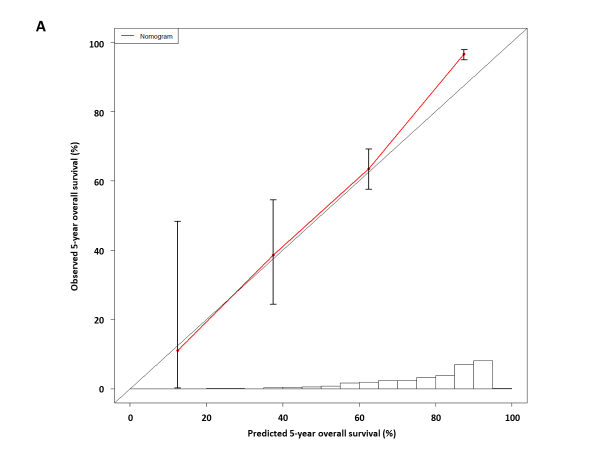


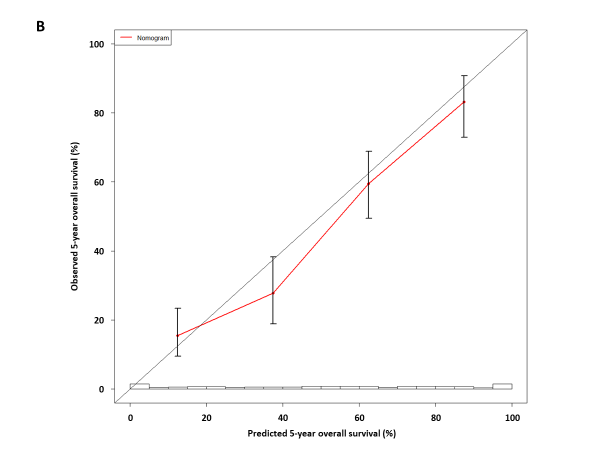


**Supporting Figure 3.** Decision curves to plot the net beneﬁt achieved by making clinical decisions based on the ﬁnal multivariable model predictions at 5 years, for overall survival.

(A) Seoul St. Mary’s Hospital validation cohort (Seoul, Republic of Korea) and (B) University of Verona validation cohort (Verona, Italy).


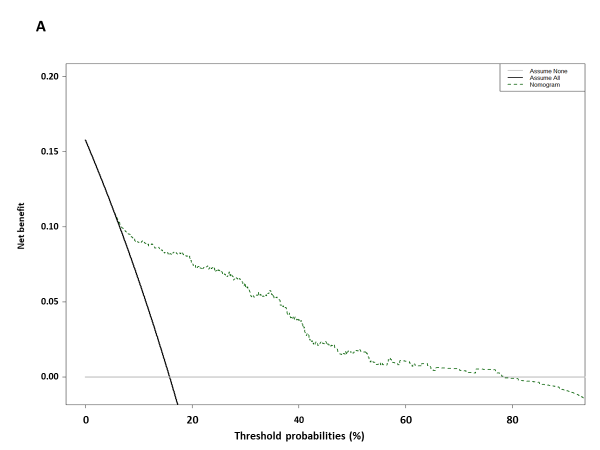


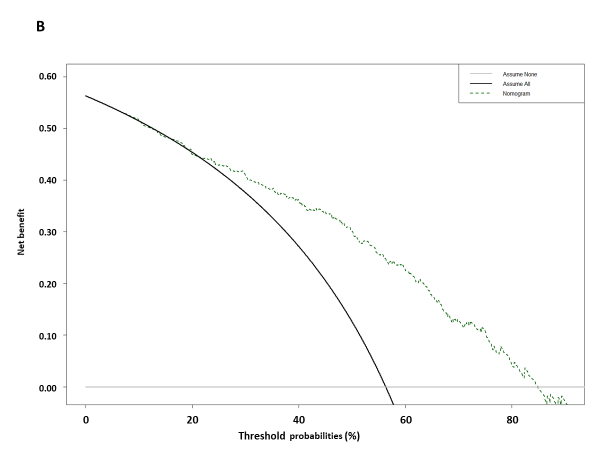


**Supporting Figure 4.** Kaplan–Meier survival curves of overall survival by American Joint Committee on Cancer (AJCC) stage grouping in each risk group in the development cohort, with the number of patients at risk.

Nomogram prediction: (A) low-, (B) intermediate-, and (C) high-risk groups


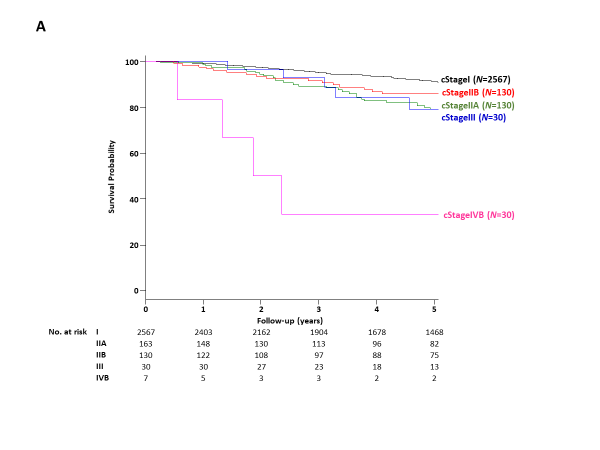


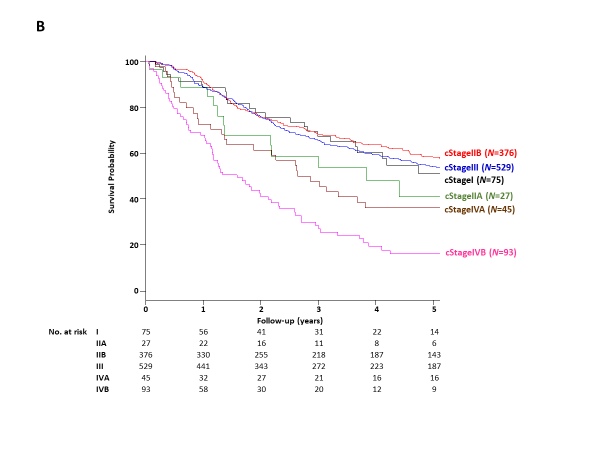


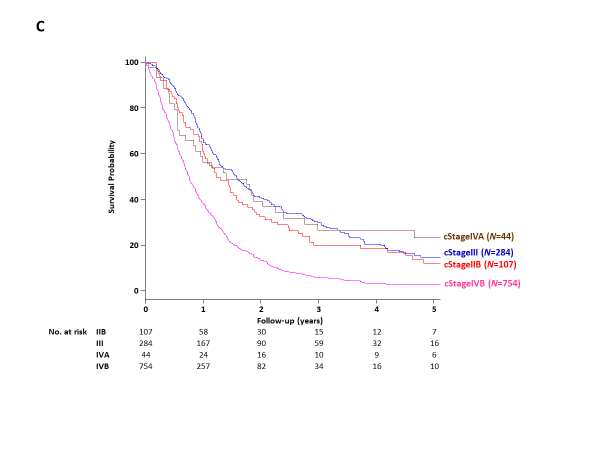


**Supporting Figure 5.** Kaplan-Meier survival curves of the overall survival of the patients stratified into three risk groups according to the nomogram-predicted 5-year survival rate in the Seoul St. Mary Hospital (Seoul, Republic of Korea) external validation cohort, with the number of patients at risk.

1. cStage I/ IIA, and (B) cStage IIB/III


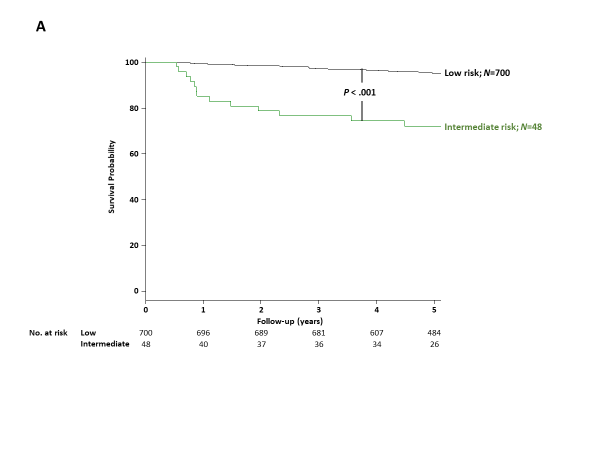


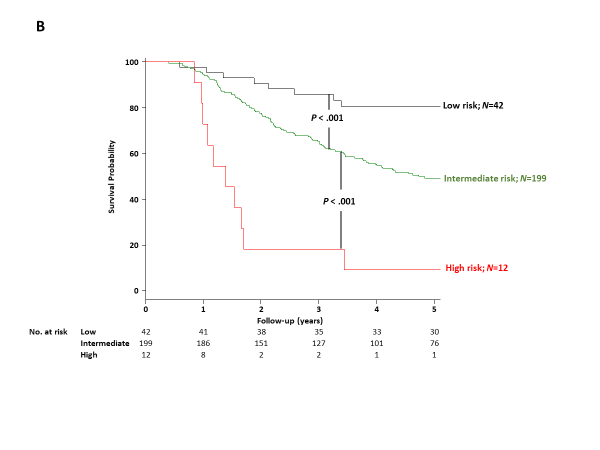


**Supporting Figure 6.** Kaplan-Meier survival curves of the overall survival of the patients stratified into three risk groups according to the nomogram-predicted 5-year survival rate in the Verona University (Verona, Italy) external validation cohort, with the number of patients at risk.

1. cStage I/IIA, (B) cStage IIB/III, and (C) cStage IVA/IVB


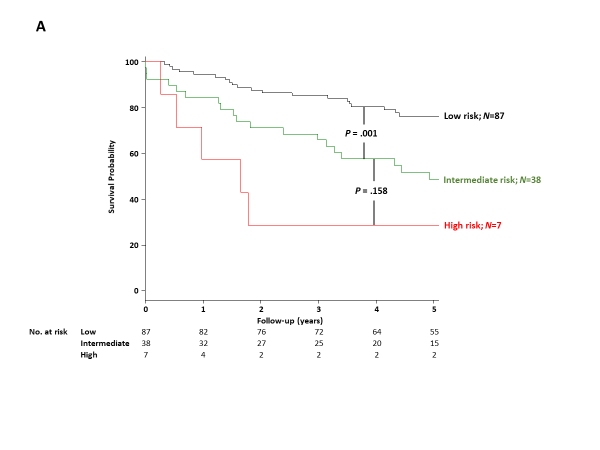


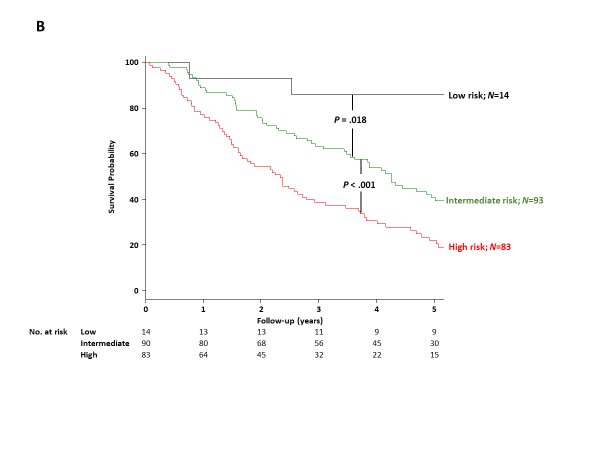


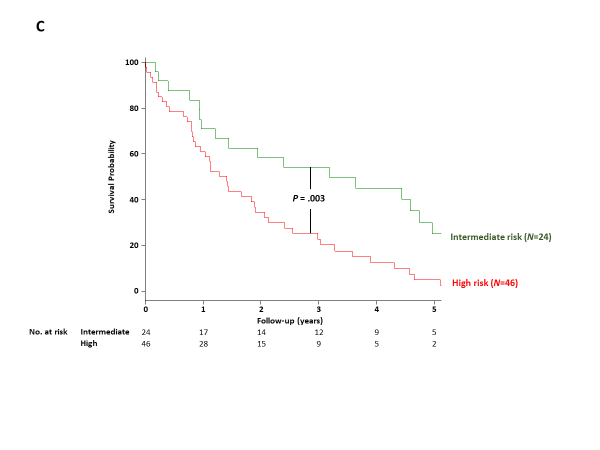


**Supporting Figure 7.** Kaplan–Meier survival curves of overall survival by American Joint Committee on Cancer (AJCC) stage grouping in each risk group in the Seoul St. Mary Hospital (Seoul, Republic of Korea) external validation cohort, with the number of patients at risk.

Nomogram prediction: (A) low-, (B) intermediate-, and (C) high-risk groups


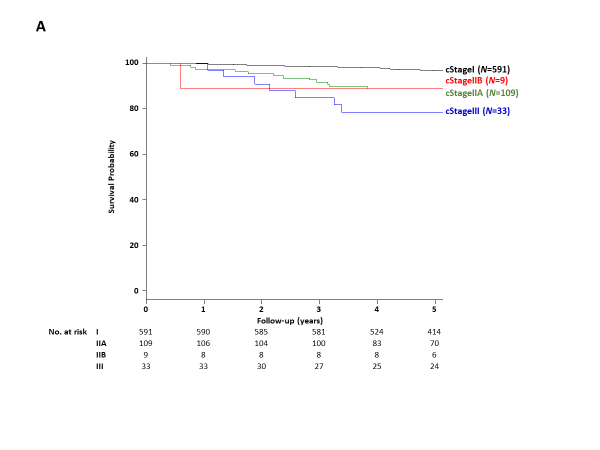


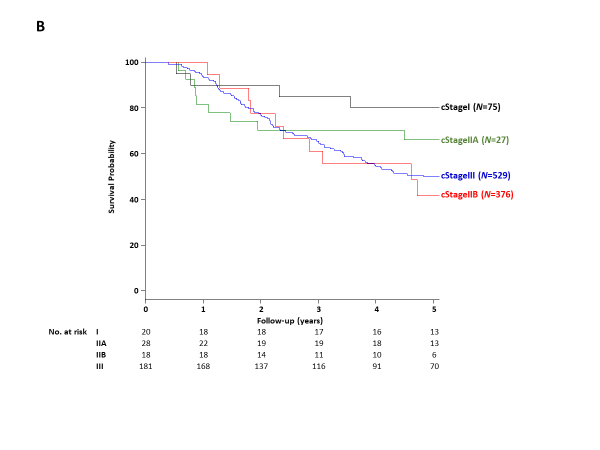


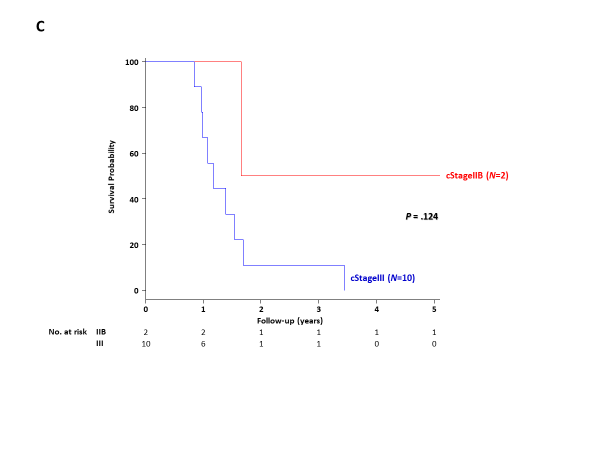


**Supporting Figure 8.** Kaplan–Meier survival curves of overall survival by American Joint Committee on Cancer (AJCC) stage grouping in each risk group in the Verona University (Verona, Italy) external validation cohort, with the number of patients at risk.

Nomogram prediction: (A) low-, (B) intermediate-, and (C) high-risk group


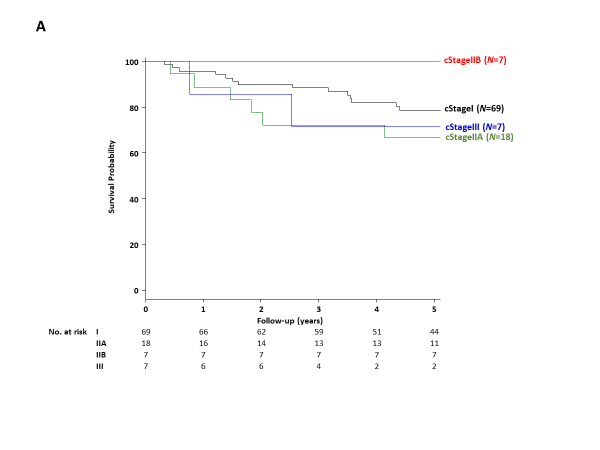


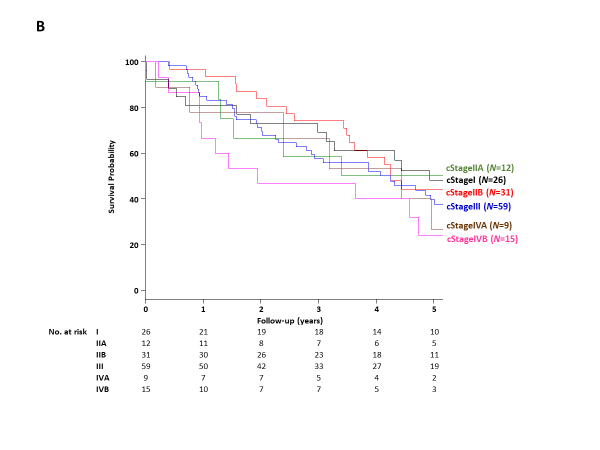


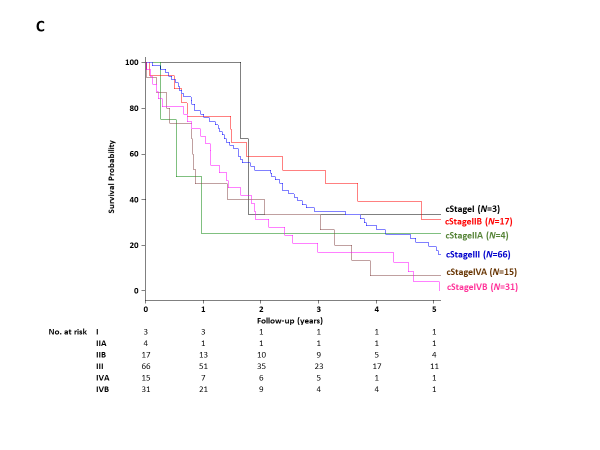

Supplement: Supplementary file 1 — DataS1 [file CAM4-9-5708-s001.docx]
